# Piloting a Genital Affirmation Surgical Priorities Scale for Trans Masculine Patients

**DOI:** 10.1089/trgh.2019.0038

**Published:** 2019-10-25

**Authors:** Oren Ganor, Amir H. Taghinia, David A. Diamond, Elizabeth R. Boskey

**Affiliations:** Center for Gender Surgery, Boston Children's Hospital, Boston, Massachusetts.

**Keywords:** bottom surgery, gender identity, metoidioplasty, phalloplasty, transgender, trans masculine

## Abstract

**Purpose:** Many transgender men seek surgical interventions to create male genitalia. Currently, there is no standardized tool to assess individual goals and expectations for such reconstructive genital surgery. The purpose of this study was to develop and pilot a genital affirmation surgical priorities scale (GASPS) in transgender men seeking metoidioplasty and/or phalloplasty.

**Methods:** The research team developed the GASPS and piloted it with 63 patients seeking reconstructive genital surgery. The scale was constructed after a comprehensive literature review identified key areas of importance, including size, erogenous and tactile sensation, interest in penetrative sex, ability to urinate standing up, and maintenance of orgasmic function. Results were then tabulated and analyzed to look for trends.

**Results:** Sixty three consecutive patients, mean age 24.98 years (standard deviation [SD]=5.87), were administered the assessment. On the 5 point Likert scale, patients were most concerned about being able to stand to urinate (mean=4.38, SD=1.06) and erotic sensation (mean=4.21, SD=0.8). The ability to engage in penetrative intercourse (mean=3.98, SD=1.34), tactile sensation (mean=3.93, SD=1.01), and penis length (mean=3.37, SD=1.18), and girth (mean=3.09, SD=1.20) were not universally considered to be important and responses varied widely. Most patients (86%) stated they had a history of being able to orgasm, and 8% did not know. Feedback suggested that scale use helped patients clarify goals for surgery.

**Conclusion:** GASPS use confirmed the diversity of patient priorities and the importance of individualized goal assessment. It also confirmed previous reports that standing to urinate is a major genital affirmation motivation for many transgender men.

## Introduction

Over the past decade, there has been a shift in the visibility and acceptance of transgender individuals. In the United States, gender-affirmation care has become much more commonly accepted and also covered by many public and private insurance plans.^1,2^ There are two major types of genital affirmation operations available for transgender men.^3^ The first, metoidioplasty, uses the hormonally enlarged clitoris as the shaft of a small neophallus. The second, phalloplasty, uses a flap to create a phallus, most commonly a radial forearm free flap.^4–7^ Urethral lengthening, which can be done as part of either phalloplasty or metoidioplasty, is also an important part of genital affirmation for many men. Extending the urethra to the tip of the neophallus gives patients the ability to urinate while standing.^8,9^

For those interested in using their neophallus for sexual penetration, phalloplasty is generally recommended over metoidioplasty. After the neophallus has healed and protective sensation has returned through the distal end of the flap, an internal penile prosthesis can be implanted to allow the phallus to become erect (although prosthesis insertion is associated with a high complication rate).^10^ With metoidioplasty, the neophallus maintains the native erectile function of the clitoris, but phallus length may not be sufficient for sexual penetration.^11^

Determining the appropriate type of genital affirmation surgery for any given patient requires a thorough understanding of their individual needs and goals, such as interest in standing urination and/or sexual penetration. There are also a variety of other factors that may affect the choice of procedure, including insurance coverage and other access concerns.^12^ Previous research has suggested that for many trans masculine individuals, the ability to urinate while standing is a major reason for seeking genital affirmation surgery.^13^ Other studies have focused more on general appearance and satisfaction with sexual function after metoidioplasty and phalloplasty.^3,14^

To date, no one has published a standardized presurgical assessment tool for trans masculine patients seeking genital affirmation surgery. Including such a tool in the broader assessment process has the potential to improve clinicians' understanding of patient goals and how likely these goals are to be met by anticipated surgical outcomes. The purpose of this study was to identify what factors should be included in such an assessment, develop a genital affirmation surgical priorities scale (GASPS), and pilot that scale on a cohort of trans masculine patients seeking genital affirmation.

## Methods

A review of the literature was performed to identify articles addressing trans masculine patients' priorities for genital affirmation surgery. An initial search was performed on PubMed using the terms “transgender and phalloplasty” and “transgender and metoidioplasty.” These articles were reviewed for information about patient priorities and preferences for genital affirmation surgery, and any articles that did not contain this information were dropped from the analysis. A list of described patient priorities was maintained as each article was analyzed, and this list was added to, as needed.

After common factors were identified across research articles, a formal assessment was created to determine trans masculine patient's genital surgery priorities. Of note, an additional question was added to the survey after the first 41 patients had been seen, to address issues brought up by the reviewers of the poster presentation on which this article was based. This study consists of an evaluation of the survey results after the expanded survey was piloted with an additional 22 patients.

The GASPS was included as part of the routine multidisciplinary assessment that was part of the initial genital surgery consultation. That assessment, which also included a standardized psychosocial readiness screening, was completed by the social worker (E.R.B.), and usually took between 30 and 45 min. Results of the psychosocial assessment, including the scale, were presented to the surgeon (O.G.) before his initial discussion with the patient.

To determine the diversity of patient responses, means and standard deviations (SDs) were calculated for each area of prioritization, and these results were graphed using box and whisker plots. Ordinal regression was used to calculate differences in prioritization of individual items by age and by the type of surgery patients expressed interest in before completing the scale using Stata 15 (StataCorp LLC, 2017, College Station, TX). This analysis plan was approved through expedited review by the Boston Children's Hospital IRB, and a separate consent was not obtained as the assessment was used as a part of clinical care.

## Results

### Literature review and scale development

To date, there has been very little research addressing trans masculine patient's goals and expectations for genital affirmation surgery.^15^ The initial literature search identified 54 articles, and 11 articles that discussed patient priorities were included in the final review. These articles included only three studies that directly assessed patient priorities, the rest consisted of expert commentaries and systematic reviews.

A concept of the “ideal neophallus” was articulated in the early research and has been broadly accepted by researchers, despite the lack of data from patients seeking genital affirmation.^3,16,17^ This ideal structure is defined as being created in a single stage, having a competent urethra, allowing for the insertion of a penile prosthesis, providing tactile and erogenous sensation, and being esthetically pleasing with minimal scarring in the donor area.^16,17^ Although some researchers have questioned the accuracy of this definition of the ideal neophallus, many analyses accept it as fact, and an assessment tool to address the assumption that there is an “ideal neophallus” has not yet been published.^18,19^

**FIG. 1.**
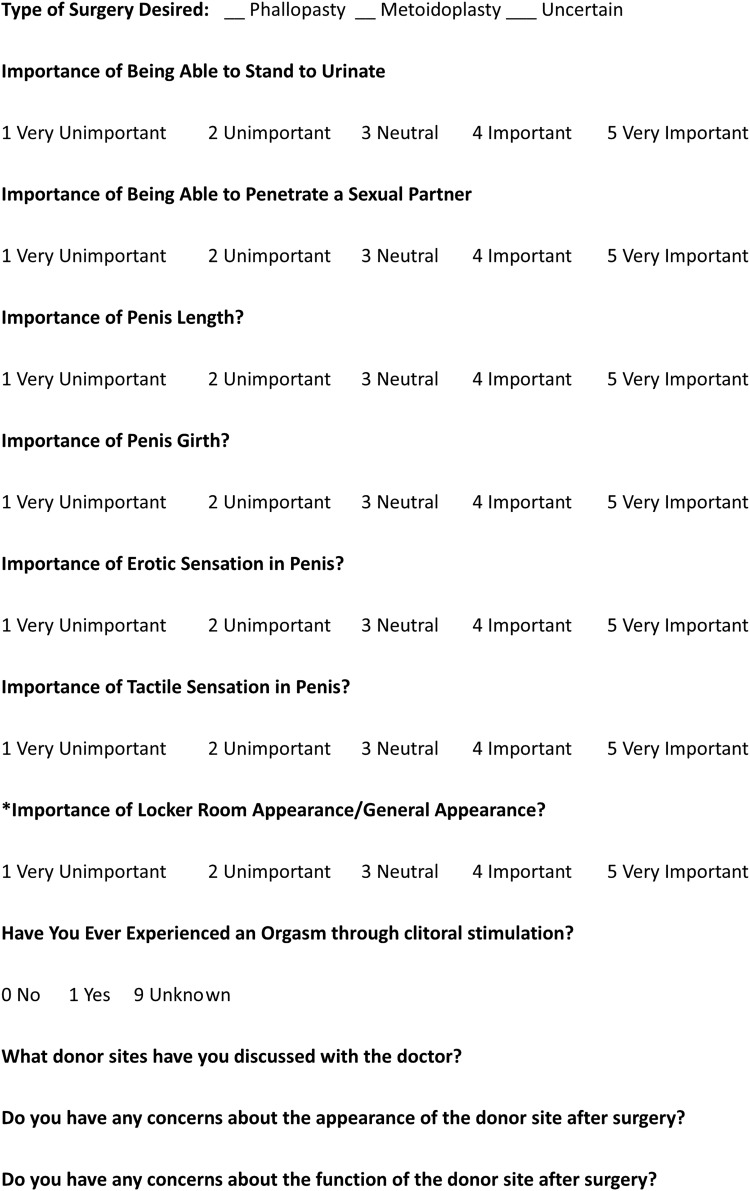
The genital affirmation surgical priorities scale. The *’d item was added in response to feedback and only piloted on the final 22 patients.

Looking at the limited data, postsurgical patients have retrospectively reported motivations of gender confirmation, appearance (including size), and standing to urinate, as well as sexual function goals, and some of those patients have expressed regret at the type of surgery that was chosen—although not at having genital surgery.^13^ These are also the areas in which postoperative satisfaction has been addressed.^3,13,14^ As such, these areas were used as the basis for the development of the initial scale, a 7-item quantitative scale with four additional open-ended questions to generate discussion about expected patient concerns, which could be used as a decision-making tool to determine both the appropriateness of a particular type of surgery and the degree to which patient expectations are realistic or not. In response to reviewer feedback, that scale was later revised to include an additional question about the importance of phallus appearance (Fig. 1). The intention for tool development was to assure consistency and thoroughness of patient priority assessment in the context of a larger clinical and social evaluation, not for the tool to be used on its own.

### Assessment results

The first 63 trans masculine patients seeking genital confirmation surgery were given the GASPS by the team social worker, as part of the standardized behavioral health assessment. No patients were excluded from the analysis. Patient ages ranged from 17 to 49 years (mean=24.98, SD=5.90). Most of the patients (86%) stated they had a history of orgasm from clitoral stimulation, with 6% and 8% stating, respectively, that they had not ever had an orgasm or did not know. All but 12 men presented initially as interested in phalloplasty, with 7 seeking metoidioplasty and 5 undecided. Several of the undecided patients stated the tool was useful in determining what genital surgery option would be most appropriate for them and, moreover, that completing the GASPS in the context of clinical care helped them understand the functional differences between the two reconstructive options. The tool also prompted open conversations about size, sexual function, and procedure concerns with the vast majority of the patients.

**FIG. 2.**
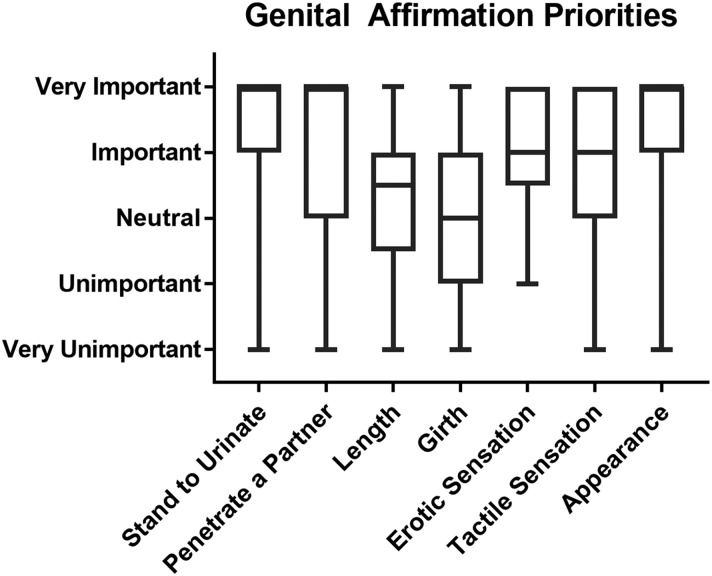
Box and whisker plot of genital affirmation priorities in trans masculine patients seeking gender-affirming surgery. The middle bar denotes the median value, the box encloses the 25th through 75th percentiles, and the whiskers extend to the minimum and maximum result. Where there is no middle bar visible, it coincides with the top edge of the box. This indicates that half or more of the results were recorded as “very important.” *N*=63 except for the appearance question, where *N*=22.

On the 5-point Likert scale, patients were most concerned about being able to stand to urinate (mean=4.38, SD=1.06) and erotic sensation (mean=4.21, SD=0.8). The ability to engage in penetrative intercourse (mean=3.98, SD=1.34), tactile sensation (mean=3.93, SD=1.01), penis length (mean=3.37, SD=1.18), and girth (mean=3.09, SD=1.20) were not universally considered to be important and responses varied widely. Finally, most patients (86%) stated that they had a history of being able to orgasm, and 8% did not know. Of the 22 patients who were asked about the importance of appearance, most patients rated it very highly (mean=4.5, SD=0.95), with very little variation (Fig. 2).

No scale variables differed significantly by age. Patients expressing an interest in phalloplasty endorsed significantly greater concerns (*p*<0.05) about penetrating a partner and penis length than the rest of the population. Patients interested in metoidioplasty endorsed significantly less concern about standing to urinate, penetrating a partner, penis length, penis girth, and tactile sensation. There was no difference in endorsement of the importance of erotic sensation for any surgery type, and across all variables, patients who were uncertain about surgery type were no different than the rest of the population (Table 1). There were too few respondents to analyze the appearance variable by interest in type of surgery. Qualitative responses were not coded for this article, but they were used as a clinical tool to stimulate discussion of patient concerns about appearance and function after surgery.

**Table 1.** Association Between Scale Variables and Type of Surgery Patients Were Interested in

	OR [95% CI]	*p* > |z|
Stand to urinate		
Phalloplasty	1.55 [0.53–4.51]	0.425
Metoidioplasty	**0.08 [0.01–0.32]**	**0.001**
Did not know	0.35 [0.07–1.81]	0.212
Penetrate a partner		
Phalloplasty	**3.44 [1.22–9.67]**	**0.019**
Metoidioplasty	**0.07 [0.01–0.42]**	**0.003**
Did not know	1.27 [0.21–7.56]	0.791
Penis length		
Phalloplasty	**3.74 [1.41–10.45]**	**0.008**
Metoidioplasty	**0.13 [0.03–0.54]**	**0.006**
Did not know	0.30 [0.06–1.57]	0.153
Penis girth		
Phalloplasty	2.26 [0.83–6.12]	0.109
Metoidioplasty	**0.20 [0.05–0.78]**	**0.020**
Did not know	0.30 [0.06–1.47]	0.137
Erotic sensation		
Phalloplasty	0.93 [0.34–2.55]	0.890
Metoidioplasty	1.52 [0.32–7.11]	0.595
Did not know	0.71 [0.13–3.83]	0.699
Tactile sensation		
Phalloplasty	2.30 [0.84–6.33]	0.106
Metoidioplasty	**0.12 [0.02–0.71]**	**0.019**
Did not know	0.19 [0.04–1.08]	0.061

Reference group for ordinal logistic regression models is the remainder of the population. Items in bold are significant at *p*<0.05.

CI, confidence interval; OR, odds ratio.

## Discussion

Trans masculine patients have a variety of goals for genital surgery, not all of which can be effectively or safely met with currently available procedures. There are numerous anecdotal reports of men choosing to delay genital affirmation surgeries due to dissatisfaction with the state of the technology and resulting outcomes.^15,20^ As such, it is important for surgical teams to have a strong understanding of patients' expectations for surgery to counsel them effectively about their options. Open and honest discussion about sexual penetration and activity can assist in directing patients to the most appropriate procedure.

The GASPS was developed to assist clinicians in working with patients to clarify their goals and expectations for genital surgery. Used as part of a broader assessment, the tool prompted in-depth conversations about surgical goals. Many of these discussions were with patients who were attempting to balance their goals for surgery with their tolerance for risk. For example, there was one patient who stated that standing to urinate was his top priority in his initial consultation, but after discussions with post-phalloplasty patients he decided that he was unwilling/unable to tolerate the high risk of urethral complications.^9,21^ This led to multiple visits where the surgical team and patient worked together to determine whether available surgical techniques would be able to give him an outcome he could be satisfied with. For many other patients, conversations focused more on their concerns about postsurgical scarring,^22–25^ and how to balance their functional goals with their esthetic preferences, in terms of both technique (metoidioplasty/phalloplasty) and flap (radial forearm/anteriolateral thigh) choice. Having a quantitative assessment of how individuals ranked specific functional issues, as well as a qualitative assessment of other concerns, allowed the team to help patients better conceptualize the risks and benefits of their various options. In addition, the ways in which our results varied by desired surgery type support the idea that patients, in general, have a good idea of which form of genital surgery will best address their needs.

To date, there has been remarkably little information published about patient goals and expectations from trans masculine genital affirmation surgeries.^3,13,15,18,20^ Analyses have largely been limited to surgical outcome reports and retrospective studies of satisfaction, which do not take individual's presurgical expectations and desires into account.^3,7,26–30^ This could potentially lead to offering a patient a surgery that is not aligned with his goals and desires but based on the surgeon's preferences. Previous researchers have noted the need for a presurgical assessment tool that could guide decision making around genital affirmation.^18^ One previous presurgical assessment has been piloted as a research tool, but not specifically with individuals seeking surgery.^15^ Our goal was to be able to use this tool as part of a broader longitudinal study of how surgical expectations and goals are related to postsurgical satisfaction and/or decision regret.

### Limitations

Our pilot study was limited by a relatively small and homogeneous population of patients seeking surgery. In addition, use of the formal assessment in our clinical care protocol was initiated before undertaking a formal validation protocol. Despite this, the observed diversity of responses reinforced the notion that it is important to assess individual goals and priorities for surgery when determining the best option for any given patient,^18,19^ rather than to assume that all patients have the same priorities and goals for their genital surgery.

One element that was not initially included in this tool, although it was included in the broader assessment, was a discussion of specific appearance priorities—such as the presence or absence of a surgically constructed glans. Appearance, sometimes conceptualized as “locker room appearance,” is a primary motivation for some men to undergo phalloplasty, and was incorporated into the tool after integration of expert feedback, and the expanded tool was used with the final 22 patients.^15^ Recent research suggests that it may also be worthwhile to assess concerns about appearance in clothing (bulkiness) vs appearance while unclothed.^15^

## Conclusion

The World Professional Association of Transgender Health Standards of Care (WPATH SOC) addresses the importance of interdisciplinary care in the assessment of readiness for genital surgery in trans masculine patients.^31,32^ The WPATH SOC also emphasize the importance of patients' understanding, and being able to choose from different surgical techniques to best accomplish their goals.^31^ We believe that including a standardized tool for trans masculine patients in a broader and more detailed assessment of surgical priorities may help both providers and patients. Based on our experience, this leads to providers and patients having these discussions in a more consistent manner, improving patient care, and hopefully reducing the risk of procedure-choice regret.^13^

## Disclaimer

Preliminary data on patient priorities for gender-affirming care (*n*=20) were initially presented at American Society of Plastic Surgeons: The Meeting on September 28, 2018.

## Abbreviations Used

CI: confidence interval
GASPS: genital affirmation surgical priorities scale
OR: odds ratio
SD: standard deviation
WPATH SOC: World Professional Association of Transgender Health Standards of Care
